# A temporal gene expression map of Chrysanthemum leaves infected with *Alternaria alternata* reveals different stages of defense mechanisms

**DOI:** 10.1038/s41438-020-0245-0

**Published:** 2020-03-01

**Authors:** Ye Liu, Jingjing Xin, Lina Liu, Aiping Song, Zhiyong Guan, Weimin Fang, Fadi Chen

**Affiliations:** State Key Laboratory of Crop Genetics and Germplasm Enhancement, Key Laboratory of Landscaping, Ministry of Agriculture and Rural Affairs, College of Horticulture, Nanjing Agricultural University, Nanjing, China

**Keywords:** Plant immunity, Gene expression

## Abstract

Chrysanthemum (*Chrysanthemum morifolium*) black spot disease (CBS) poses a major threat to Chrysanthemum cultivation owing to suitable climate conditions and current lack of resistant cultivars for greenhouse cultivation. In this study, we identified a number of genes that respond to *Alternaria alternata* infection in resistant and susceptible Chrysanthemum cultivars. Based on RNA sequencing technology and a weighted gene coexpression network analysis (WGCNA), we constructed a model to elucidate the response of Chrysanthemum leaves to *A. alternata* infection at different stages and compared the mapped response of the resistant cultivar ‘Jinba’ to that of the susceptible cultivar ‘Zaoyihong’. In the early stage of infection, when lesions had not yet formed, abscisic acid (ABA), salicylic acid (SA) and EDS1-mediated resistance played important roles in the Chrysanthemum defense system. With the formation of necrotic lesions, ethylene (ET) metabolism and the Ca^2+^ signal transduction pathway strongly responded to *A. alternata* infection. During the late stage, when necrotic lesions continued to expand, members of the multidrug and toxic compound extrusion (MATE) gene family were highly expressed, and their products may be involved in defense against *A. alternata* invasion by exporting toxins produced by the pathogen, which plays important roles in the pathogenicity of *A. alternata*. Furthermore, the function of hub genes was verified by qPCR and transgenic assays. The identification of hub genes at different stages, the comparison of hub genes between the two cultivars and the highly expressed genes in the resistant cultivar ‘Jinba’ provide a theoretical basis for breeding cultivars resistant to CBS.

## Introduction

The production of cut Chrysanthemum flowers growing under greenhouse conditions is threatened by many diseases. *Alternaria alternata*, a common necrotrophic fungus that is ubiquitously present on various plant species, damages plant tissues by producing toxins^1,2^. Warm and humid environmental conditions lead to the spread of diseases caused by this fungus, which is a year-round problem for Chrysanthemum production in greenhouses. Although the early stage of the infection is symptomless, small round black spots form at the site of *A. alternata* invasion, and these spots eventually expand into round, round-like, or irregular spots that are covered with a dark mildew layer. The widespread occurrence of this disease severely affects the yield and ornamental value of Chrysanthemum, causing substantial losses in flower production.

Currently, Chrysanthemum black spot disease (CBS) is controlled by fungicides; however, residues and severe environmental pollution caused by fungicides contradict the green, sustainable development of agricultural production. Therefore, screening and breeding resistant Chrysanthemum cultivars is the most environmentally friendly approach to controlling CBS. Furthermore, revealing the molecular mechanism of CBS resistance will provide a strong theoretical basis for formulating disease resistance breeding strategies and preventing black spot disease. In our laboratory, a variety of Chrysanthemum germplasm resources with different levels of resistance to black spot disease are being used to identify key genes involved in disease resistance.

The response of the plant immune system to pathogen infection involves two branches: PTI (pathogen-associated molecular pattern-triggered immunity) and ETI (effector-triggered immunity)^3^. PTI is mainly triggered by the perception of microbial proteins, leading to the deposition of callose in the cell wall, MAP kinase cascade signal transduction, changes in gene expression, closure of stomata, and/or an oxidative burst^4–6^. ETI is regulated by resistance genes (R genes). Activation of R genes leads to reactive oxygen production and the hypersensitive response (HR), which is considered a programmed cell death (PCD) response to impede expansion of the pathogen^7–9^. During the interaction between plants and pathogens, R genes play significant roles in the HR; the SA-dependent defense signaling pathway is invoked when the effector is recognized by an R gene^10,11^. SA signaling is also an important aspect of the PAMP response and R gene-mediated resistance^12,13^. Both ET and jasmonic acid, which are plant hormones, are crucial for the PCD response^14–17^. Additionally, abscisic acid (ABA) plays a positive role in early-acting defenses, such as stomatal closure, but plays a negative role in later-acting defense mechanisms mediated by SA or JA^18–20^. These immune responses are controlled by the reprogramming of a considerable number of defense-related genes, including those encoding transcription factors (TFs)^21,22^.

The plant immune response is closely related to the calcium ion (Ca^2+^) signal transduction pathway via the decoding of the Ca^2+^ signatures triggered by pathogens^23–27^. Different Ca^2+^ sensors have been reported to recognize Ca^2+^ signatures, such as calmodulin-like proteins (CMLs) and calmodulin (CaM), demonstrating that the conversion of Ca^2+^ signals to plant immune responses is mediated by different channels and machineries^28^. As a general secondary messenger, Ca^2+^ participates in different types of cellular processes and has important functions in sensing microbe signals to establish appropriate immune and symbiotic responses, including the production of ROS and NO as well as PCD^26^. Moreover, Ca^2+^ signaling is connected to other hormone-mediated pathways. In guard cells, stomatal closure in response to increased levels of Ca^2+^ is impaired in *cpk5/6/11/23* mutants treated with ABA^29^. Research also shows that Ca^2+^ is involved in the ET-mediated pathogenesis response^30^. Moreover, many TFs have been identified as being involved in calcium signaling. One class of TFs comprising Ca^2+^/CaM-regulated members is the WRKY family; WRKY43, WRKY53, and all members of the WRKYIId subfamily have been found to interact with Ca^2+^/CaM^31^. Although the Ca^2+^ signal transduction pathway has been a popular research topic for many years, it remains unknown whether Ca^2+^ is involved in Chrysanthemum-*A. alternata* interaction.

RNA sequencing technology is an efficient method for revealing genetic diversity and comparing variation in global expression of coding genes among genotypes/cultivars. Several transcriptome studies involving various genotypes/cultivars have been performed to understand stress responses^32^. Nevertheless, no such detailed analysis has been performed to elucidate the molecular regulatory mechanisms underlying the response of both resistant and susceptible Chrysanthemum cultivars to *A. alternata* infection.

Here, we dissect RNA sequencing time series data to reveal the transcriptional regulatory network in Chrysanthemum leaves after *A. alternata* inoculation. Our integrated analysis revealed that EDS1-mediated disease resistance and hormone and Ca^2+^ signal transduction pathways are of vital importance in the response of Chrysanthemum to infection of this fungus. Differentially expressed gene (DEG) and coexpression network analyses revealed a series of hub genes that play key roles at different stages of *A. alternata* infection. To verify the function of these hub genes, we performed transgenic experiments with *CmWRKY33.1* and acquired sense-expression genetic transformation lines. Inoculation assays showed that three lines overexpressing *CmWRKY33.1* exhibited enhanced susceptibility to black spot disease compared with that of wild-type ‘Jinba’. Our research presents insight into the molecular regulatory mechanisms underlying Chrysanthemum responses to *A. alternata* inoculation.

## Results

### Evaluation of Chrysanthemum cultivars after inoculation with *A. alternata*

**Fig. 1:**
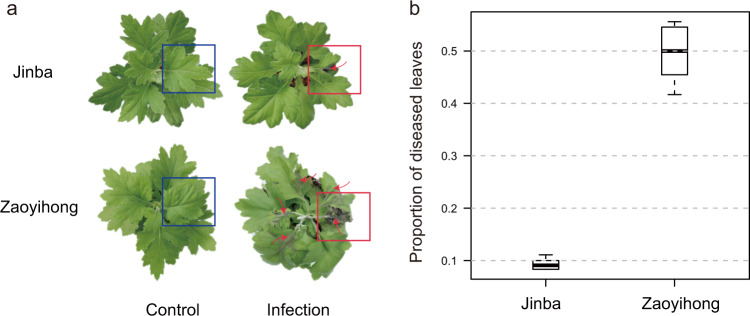
Evaluation of the response of two Chrysanthemum cultivars to Chrysanthemum black spot disease (CBS). **a** Images of the resistant cultivar ‘Jinba’ and the susceptible cultivar ‘Zaoyihong’ with CBS. **b** Proportion of diseased leaves of *A. alternata* infections in ‘Jinba’ and ‘Zaoyihong’

We selected two Chrysanthemum cultivars, ‘Jinba’ (resistant cultivar) and ‘Zaoyihong’ (susceptible cultivar), which differ significantly in their symptoms after *A. alternata* inoculation. The black leaf spot disease symptoms of the two Chrysanthemum cultivars were investigated at 3 days post inoculation. ‘Jinba (SM)’ displayed necrotic lesions that were mostly limited to the inoculated leaf, whereas ‘Zaoyihong (ZYH)’ exhibited necrotic lesions on other leaves, including the leaf inoculated with *A. alternata* (Fig. 1a). The disease symptoms were evaluated based on the proportion of diseased leaves at 3 days post inoculation; as shown in Fig. 1b, the proportions of diseased ‘Jinba’ and ‘Zaoyihong’ leaves were 0.08–0.10 and 0.45–0.56, respectively.

Leaves inoculated with *A. alternata* were sampled at six time points (0 h, 1 h, 6 h, 12 h, 24 h, and 36 h) based on the phenotypes to analyze differences in transcript levels between ‘Jinba’ and ‘Zaoyihong’, with three biological replicates sampled for each cultivar at each time point.

### Generation of transcriptomic time series data via RNA sequencing

RNA sequencing technology is an effective method for revealing genetic diversity and comparing the variation in global expression of coding genes among various genotypes/cultivars. To further investigate the transcriptome dynamics and regulatory mechanism of Chrysanthemum leaf responses to *A. alternata*, we performed a comparative transcriptome-wide time series analysis of the two Chrysanthemum cultivars, ‘Zaoyihong (ZYH)’ and ‘Jinba (SM)’, with three independent biological replicates for all samples at each time point. In total, 36 cDNA libraries from the leaf tissues of the two cultivars were generated: SM_0_1, SM_0_2, SM_0_3, SM_1_1, SM_1_2, SM_1_3, SM_6_1, SM_6_2, SM_6_3, SM_12_1, SM_12_2, SM_12_3, SM_24_1, SM_24_2, SM_24_3, SM_36_1, SM_36_2, SM_36_3, ZYH_0_1, ZYH_0_2, ZYH_0_3, ZYH_1_1, ZYH_1_2, ZYH_1_3, ZYH_6_1, ZYH_6_2, ZYH_6_3, ZYH_12_1, ZYH_12_2, ZYH_12_3, ZYH_24_1, ZYH_24_2, ZYH_24_3, ZYH_36_1, ZYH_36_2, and ZYH_36_3. The raw data were then converted to clean reads, which were subsequently used for further analysis (Table S1). As a result, 1.7 billion clean reads (average of ~23.3 million reads from each sample) were obtained. All clean data can be downloaded from NCBI (BioProjects: PRJNA596929).

### Analysis of DEGs in the time series samples

RSEM and BOWTIE were used to process the clean reads for each sample to obtain the expected FPKM (fragments per kilobase of transcript length per million mapped reads) number for each gene. The DESeq R package was used to identify differentially expressed genes. Pearson correlation analysis was then applied to compare the RNA sequencing data of the three biological replicates, which varied from 0.87 to 0.93, indicating high consistency among the replicates (Fig. S1).

**Fig. 2:**
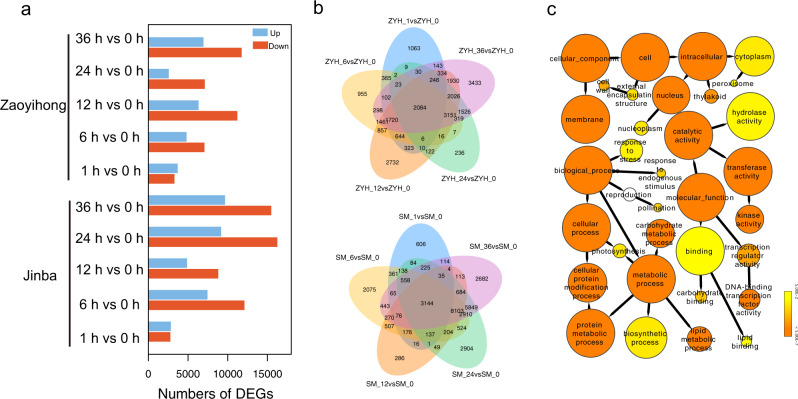
Statistical and GO analyses of DEGs in ‘Jinba’ and ‘Zaoyihong’ after infection with *A. alternata*. **a** Statistical analysis of upregulated and downregulated differentially expressed genes (DEGs) in response to *A. alternata* infection. **b** Venn diagrams of upregulated and downregulated differentially expressed genes (DEGs) in response to *A. alternata* infection in ‘Zaoyihong’ (top) and ‘Jinba’ (bottom). **c** GO analysis of DEG responses to *A. alternata* infection in ‘Jinba’ and ‘Zaoyihong’. The enriched categories are represented by circles, and the size of each circle represents the number of genes

To investigate the temporal dynamics of transcriptional reprogramming between the two Chrysanthemum cultivars following challenge with *A. alternata*, we plotted the number of DEGs (padj <0.05) against those of noninoculated plants (SM_0 or ZYH_0) at each inoculation time point. In the ‘Jinba’ cultivar, 5740 (2835 upregulated; 2905 downregulated), 19,691 (12,176 upregulated; 7515 downregulated), 13,805 (8868 upregulated; 4937 downregulated), 25,549 (16,315 upregulated; 9234 downregulated) and 25,375 (15,543 upregulated; 9732 downregulated) DEGs were found in the samples at the five inoculation times; 7104 (3352 upregulated; 3752 downregulated), 12,010 (7129 upregulated; 4881 downregulated), 17,662 (11,277 upregulated; 6385 downregulated), 9813 (7172 upregulated; 2641 downregulated) and 18,826 (11,816 upregulated; 7010 downregulated) DEGs were identified in ‘Zaoyihong’ (Fig. 2a). The largest number of stage-specific genes (25,549 in ‘Jinba’ and 18,826 in ‘Zaoyihong’) was identified in the comparison of SM_24 and ZYH_36 compared with that of SM_0 and ZYH_0 (Fig. 2a). In total, 39,802 DEGs were obtained from the two cultivars; of these, 2084 DEGs were shared among the five time series stages in ‘Jinba’, and 3144 DEGs were shared in ‘Zaoyihong’. We also identified DEGs uniquely expressed at each time series stage, as shown in Fig. 2b, and the results suggest that each stage has its own independent defense mechanisms.

The results of our study showed that a large number of DEGs, up to 33,343 in the resistant cultivar ‘Jinba’ and 26,173 in the susceptible cultivar ‘Zaoyihong’, respond to *A. alternata* infection. This is a considerably greater number of genes than previously reported in similar transcriptomic studies. By using BINGO, we next performed GO analysis to further characterize the identified DEGs. As shown in Fig. 2c, the DEGs were distributed among the three GO categories: “biological process”, “molecular function”, and “cellular component”. The DEGs enriched in the “cellular component” category are mainly involved in “cell wall”, “peroxisome”, “nucleoplasm”, and “thylakoid”. Enriched terms in the “biological process” category mainly included “response to stress”, “response to endogenous stimulus”, and “carbohydrate metabolic process”. The GO terms “hydrolase activity”, “kinase activity”, “DNA-binding transcription factor activity”, “carbohydrate binding”, and “lipid binding” were enriched in the “molecular function” category.

### Pathway analysis of Chrysanthemum in response to *A. alternata* infection

To facilitate our analysis of the plant and fungal networks, we also mapped the pathways of the DEGs involved in Chrysanthemum-*A. alternata* interactions using the KEGG database (www.genome.jp/kegg). Plant hormones are crucial for helping plants adapt to adverse environmental conditions. Our data showed that the expression of genes involved in hormone signaling, especially that of ET and ABA, was upregulated when Chrysanthemum leaves were challenged with *A. alternata* (Fig. S2b and c); the expression of a small proportion of genes involved in other hormone signal transduction pathways was also upregulated (Fig. S3). Traditionally, it has been thought that mainly ET and JA respond to necrotrophic fungi, but SA mediates defense signaling against both hemibiotrophic and biotrophic fungi. Based on our data, SA might also play a significant role in local immunity against necrotrophic fungi (Fig. S3). Moreover, plant-pathogen interaction pathway analysis indicated that the expression of genes involved in the Ca^2+^-dependent signaling pathway was upregulated by more than 2-fold (Fig. S2a).

The transcriptional changes in the leaves of resistant and susceptible Chrysanthemum cultivars infected with *A. alternata* were also visualized via MapMan software. Figure S4 depicts the general overview of the cellular response, confirming that the number of DEGs responding to biotic stress was greater than the number responding to other stresses. Figure S5 illustrates a general overview of the biotic stress response. MapMan visualization revealed that many DEGs are involved in cell wall strengthening, transcriptional regulation, the oxidative respiratory burst, cellular signaling, and hormone metabolism. Upregulated genes play important roles in the response to pathogen infection. Moreover, the expression of most of the DEGs related to ET metabolism, the respiratory burst and mitogen-activated protein kinases (MPKs) was upregulated at different times after *A. alternata* inoculation, which suggests the importance of these pathways for the resistance of Chrysanthemum to infection by this fungus.

In total, the involvement of multiple signal transduction pathway genes suggests that the response of Chrysanthemum leaves to *A. alternata* infection is not simply linear cascades but rather is a combination of multiple signaling pathways. Previous studies have shown that ET and the H_2_O_2_-mediated HR and PCD play vital roles in sand pear–*A. alternata* interaction^33^. In our study, we identified that ET, ABA, and Ca^2+^ are important in the defense response to *A. alternata* inoculation and highlight the regulatory mechanism occurring in Chrysanthemum leaves.

### Identification of gene coexpression modules during *A. alternata* infection

WGCNA is a powerful tool for identifying a set of genes associated with phenotypes. To investigate the gene regulatory network of Chrysanthemum leaves in response to *A. alternata* infection, we performed a WGCNA by using RNA sequencing profiles. After the expression data were preprocessed, a total of 36,403 genes were subjected to WGCNA, and we ultimately discovered 14 gene coexpression modules. The number of genes in these modules ranged from 62 to 8235.

**Fig. 3:**
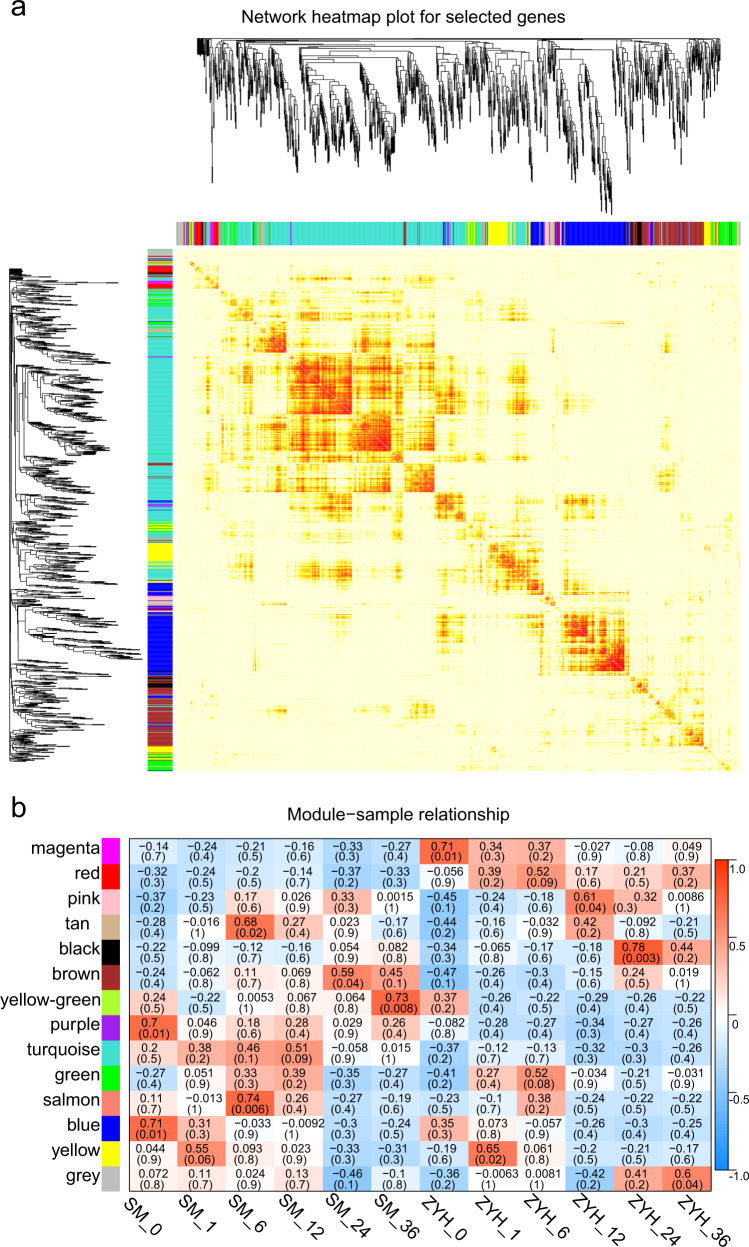
WGCNA of the transcripts in ‘Jinba’ and ‘Zaoyihong’. **a** Dendrogram of genes based on a coexpression network analysis in ‘Jinba’ and ‘Zaoyihong’. Each leaf in the tree corresponds to an individual gene. Fourteen modules, each associated with a different color, formed from the major tree branches. **b** Association between modules and traits. The color of each module is the same as that in **a**. Each column represents a leaf necrotic lesion trait. The legend indicates the correlation. Each cell contains the number of corresponding correlations and *P* values

The eigengenes for the 14 modules correlated with different infection stages (Fig. 3). We studied the preservation of coexpression modules between ‘Jinba’ and ‘Zaoyihong’ at different stages of *A. alternata* infection via module-trait relationship and module-sample relationship analyses. In most of the modules, the trend of genes was nearly the same between the two cultivars at different inoculation times, as indicated by the yellow, tan, and brown colors in the figure. Interestingly, a few modules differed in response to *A. alternata* infection. Notably, we identified one module of ‘Jinba’, i.e., that associated with the turquoise color in the figure, that was cultivar specific.

### Transcriptional regulatory modules associated with the expansion of necrotic lesions

We investigated the transcriptome of Chrysanthemum leaves infected with *A. alternata* and collected samples at six time points. The early stage of infection was symptomless up to ~1 h post inoculation (hpi). The first signs of necrosis were observed at 6 hpi, and round or irregular lesions formed at 12 hpi at the inoculation sites, which was considered the middle stage/period of infection. During the late stage of infection, the lesions expanded to the outside of the inoculation site at 24 hpi, and half of the Chrysanthemum leaves showed black necrotic spots at 36 hpi. Plant defense genes play crucial roles during fungal infection, and the expression of many is regulated by TFs. Therefore, we further analyzed the important defense genes and TFs with a strong regulatory relationship with the coexpressed defense genes in these notable modules.

**Fig. 4:**
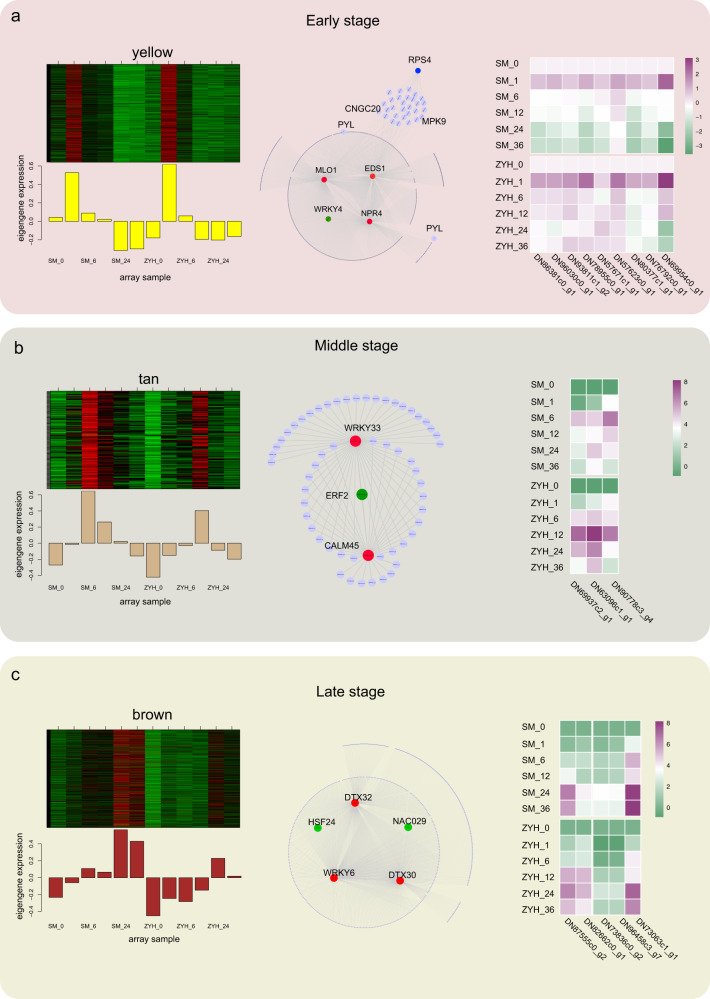
DEGs at different stages of the response of Chrysanthemum leaves to *A. alternata* infection. **a** Coexpression network of the yellow module. **b** Coexpression network of the tan module. **c** Coexpression network of the brown module. The coexpressed genes in each module are shown in heatmaps and bar graphs (left). The red rectangles in the heatmap represent high expression; the green rectangles represent low expression. The network of top hub genes is indicated by larger circles and the red color in the network (middle). Transcription factors with strong regulatory relationships (weight >0.3) in the yellow modules are indicated by larger circles and green color in the network. The dots around the circle represent other coexpressed genes indicated by small dots, and the relationships are connected by lines. The heatmaps (right) show the expression of the top hub genes and transcription factors that present strong regulatory relationships with the top hub genes

Combined with our WGCNA, we identified key genes (also called hub genes) at different infection stages, as shown in Fig. 4. The expression of the genes in the yellow module were most upregulated at 1 hpi, corresponding to the early stage of the response of Chrysanthemum leaves to *A. alternata* infection. At 6–12 hpi, there was one tan module in which the gene expression showed peak upregulation at 6 hpi or 12 hpi. Many genes with a transcriptional peak at 24 hpi or 36 hpi were classified into the black or brown module; at 24 hpi or 36 hpi, the late stage, the lesions expanded outside of the inoculation site. In the yellow module, three hub genes, e.g., an *EDS1* homolog (*CmEDS1*; *DN78955c0_g1*), an *MLO1* homolog (*CmMLO1*; *DN86381c0_g1*), and an *NPR4* homolog (*CmNPR4*; *DN80377c1_g1*), related to defense were identified. Because TFs play important roles in gene regulation and plant immunity, we further investigated their regulatory network. TFs showed a strong regulatory relationship (weight/topological overlap matrix (TOM)>0.3) with other genes in the module and were used for further analysis. The identified *WRKY4* homolog showed a regulatory relationship with the three hub defense response genes. In addition, the expressions of the *RPS4* homolog and *PYL4* homolog were upregulated in the yellow module (Fig. 4a and Fig. S2c). In the tan module, we identified two hub genes related to plant-pathogen interactions: a *WRKY33* homolog (*CmWRKY33*; *DN63096c1_g1*) and a *CML45* homolog (*CmCML45*; *DN69937c2_g1*). An *ERF2* homolog (*CmERF2*; *DN90778c3_g1*), whose product plays important roles in the ET signal transduction pathway, was found to have a strong regulatory relationship with the two hub genes (*CmWRKY33* and *CmCML45*) (Fig. 4b). In the late stage of the Chrysanthemum response to *A. alternata* infection, we identified two hub genes belonging to the MATE family, which are involved in multiple drug and toxic compound extrusion; a *DTX30* homolog (*CmDTX30*; *DN96458c3_g7*); and a *DTX32* homolog (*CmDTX32*; *DN73836c0_g2*). One TF, a *WRKY6* homolog (*CmWRKY6*; *DN87555c0_g2*), was also identified as a hub gene. Furthermore, an *HSF24* homolog (*DN82662c0_g1*) and a *NAC029* homolog (*DN73063c1_g1*) exhibited strong regulatory relationships with other genes in the brown module, which also had regulatory relationships with two hub genes (*CmDTX30* and *CmDTX32*) (Fig. 4c). These results suggest a previously unknown function for MATE family genes in response to *A. alternata* infection.

### Coexpression networks reveal that the highly resistant cultivar ‘Jinba’ uniquely responds to *A. alternata* infection

**Fig. 5:**
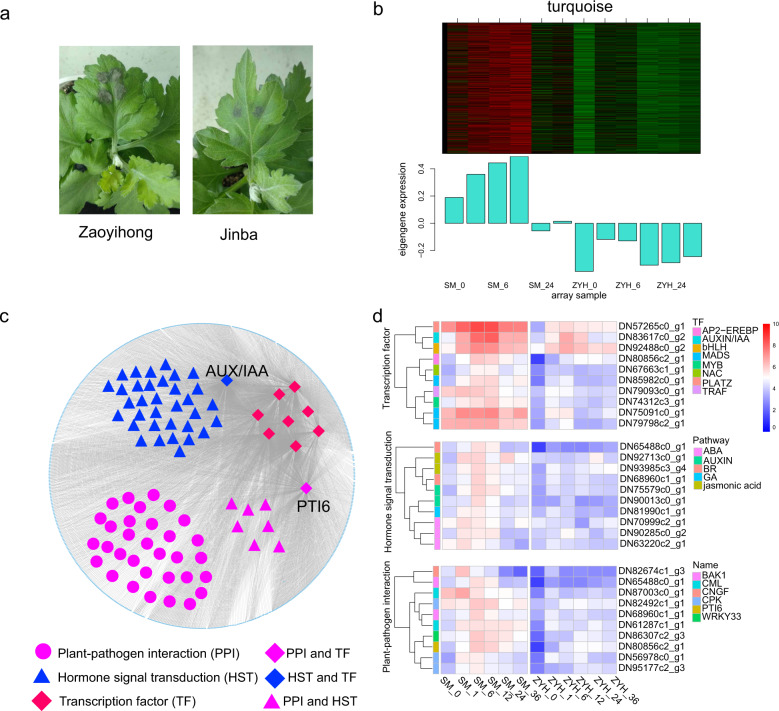
Analysis of highly expressed genes in the resistant cultivar ‘Jinba’. **a** Phenotype of Chrysanthemum leaves at 12 h post inoculation in ‘Jinba’ and ‘Zaoyihong’. **b** The coexpressed genes are shown in heatmaps and bar graphs in the turquoise module. The red rectangles in the heatmap represent high expression; the green rectangles represent low expression. **c** The turquoise module was visualized by Cytoscape. Differently expressed transcription factors are indicated in larger red, blue, or purple quadrangles. Larger purple circles, triangles, or quadrangles represent differentially expressed genes involved in the plant-pathogen interaction pathway. Differentially expressed genes involved in the hormone signal transduction pathway are indicated in larger blue or purple triangles and larger blue quadrangles. **d** Heatmaps showing the expression profile of DEGs involved in PPI and HST and transcription factors in the turquoise module. The color represents the expression levels of genes in the module as shown in the figure legend

The Chrysanthemum cultivar ‘Jinba’ was more resistant and had smaller necrotic lesions than ‘Zaoyihong’ did during infection with *A. alternata* (Fig. 5a). The ‘turquoise’ module (*r* = 0.83, *p* = 9e-04), which included 8228 genes, was cultivar specific and upregulated after *A. alternata* infection (Fig. 5b), with a possible association with the resistant phenotype.

The genes of this module were examined for further analysis. Based on the DEG analysis, we found 10 hub genes belonging to different TF families, e.g., *AP2-EREBP*, *AUX*, *bHLH*, *MADS*, *MYB*, *NAC*, *PLATZ,* and *TRAF*, to be differentially expressed (Fig. 5d). A total of 52 genes that interact with TFs according to the WGCNA are involved in plant-pathogen interactions, including *DN80856c2_g1*. Interestingly, *DN80856c2_g1*, called *PTI6*, which belongs to the *AP2-EREBP* TF family, also plays significant roles in plant-pathogen interactions. The results showed that the hub TFs may interact with genes involved in plant-pathogen interactions. Additionally, among the genes interacting with the hub TFs, 43 are involved in the hormone signal transduction pathway, which may be crucial in resistant cultivars challenged with *A. alternata*. The regulatory relationship was visualized with Cystoscope software, as depicted in Fig. 5c. Analysis of the DEGs interacting with TFs revealed one ABCA3 family gene (*DN91413c1_g3*) and four MATE family genes (*DN94014c1_g1*; *DN90910c1_g1*; *DN95310c2_g2* and *DN82887c0_g1*) that were distinctly highly expressed in the resistant cultivar ‘Jinba’ (Fig. 5d and Table S3), suggesting a previously unknown function in resistance to pathogen infection.

### Validation of DEGs

To verify the RNA sequencing data, we selected 10 DEGs from different modules for quantitative real-time PCR (qPCR) assays; the housekeeping gene *EF1α* was used as an internal control. The DEGs included *CmCML45* (*DN69937c2_g1*), *CmWRKY33* (*DN63096c1_g1*), *CmERF2* (*DN90778c3_g1*), *CmNAC029* (*DN73063c1_g1*), *CmMLO6* (*DN77454c0_g1*), *CmMYB15* (*DN77779c0_g1*), *CmHSF24* (*DN82662c0_g1*), *CmDTX24* (*DN81968c0_g1*), *CmFTSH* (*DN87341c1_g3*), and *CmVTE3* (*DN93367c1_g4*). The final qPCR data for the 10 DEGs were in accordance with the results of the transcriptome analysis, as presented in Fig. S6. Additionally, the qPCR and RNA sequencing results showed consistency according to a correlation analysis. The Pearson correlation coefficients of the results between the qPCR and RNA sequencing were 0.80–1 and 0.77–1 for the genes of ‘Jinba’ and ‘Zaoyihong’, respectively, at different stages of disease lesion development (Fig. S7), which also indicates that the data produced via RNA sequencing are reliable.

### Overexpression of *CmWRKY33.1* resulted in enhanced susceptibility to black spot disease

**Fig. 6:**
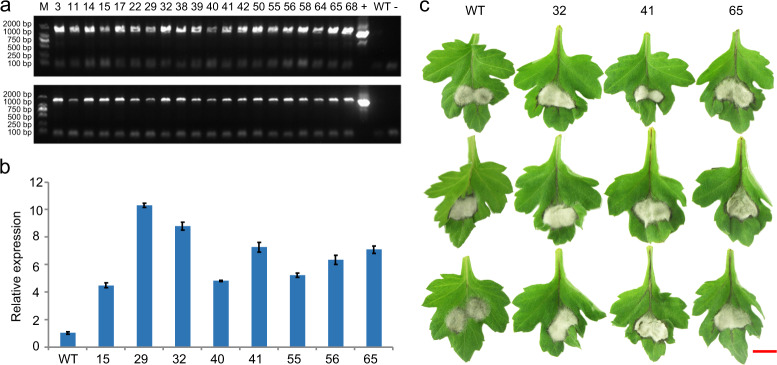
Detection of CmWRKY33.1 transgenic lines and phenotypes after inoculation with *A. alternata*. **a** Electrophoresis detection of transgenic lines. 3–68: Overexpressing lines; + Positive control (Plasmid DNA), − Negative control (H_2_O), WT Wild-type ‘Jinba’. Up: PCR primers CmWRKY33.1-ID-F1/R1 were used for amplification of the pMDC43-CmWRKY33.1 vector in the transformation lines. Down: PCR primers CmWRKY33.1-ID-F2/R2 were used for amplification of the pMDC43-CmWRKY33.1 vector in the transformation lines. **b** Relative expression of *CmWRKY33.1* in the overexpression lines. **c** Phenotypic differences of wild-type and *CmWRKY33.1*-overexpressing lines. Bar = 10 mm

In our study, we found that *WRKY* family genes were highly expressed as the lesion spread. Based on the *WRKY33* homologous sequences from our transcriptome data, we cloned one *WRKY33* homologous gene: *CmWRKY33.1*. To further verify the function of *CmWRKY33.1*, we constructed a pMDC43-*CmWRKY33.1* expression vector for genetic transformation of Chrysanthemum and acquired sense-expression transformed lines. Electrophoresis detection assays showed that the vector was successfully transformed into wild-type ‘Jinba’ (Fig. 6a), and according to qPCR assays, the expression of *CmWRKY33.1* in the transformed lines significantly increased (Fig. 6b). Moreover, inoculation assays demonstrated that, compared with wild-type ‘Jinba’, the three transformation lines overexpressing *CmWRKY33.1* displayed enhanced susceptibility to black spot disease (Fig. 6c).

## Discussion

The molecular mechanisms underlying the defense system of Chrysanthemum leaves in response to biotic stress are poorly understood. In our research, we identified a series of genes that respond to *A. alternata* at different times after inoculation in both susceptible and resistant Chrysanthemum cultivars and aimed to elucidate the mechanism by which the resistant cultivar ‘Jinba’ specifically engages its defense systems. RNA sequencing was applied to detect dynamic changes in the transcriptome of two Chrysanthemum cultivars (the resistant cultivar ‘Jinba’ and the susceptible cultivar ‘Zaoyihong’) and to investigate the molecular mechanism underlying lesion expansion. Analysis of the transcriptome time series and WGCNA revealed that pathogen-induced stress triggers defense responses in Chrysanthemum. Our research thus elucidated how Chrysanthemum conveys various signals in response to *A. alternata* infection.

When no lesions formed on Chrysanthemum leaves after *A. alternata* inoculation, the defense regulator *CmEDS1* plays significant roles in the response to infection, which is consistent with the involvement of *CmRPS4*, *CmNPR4*, and *CmPYL4*. In *Arabidopsis*, EDS1 together with RPS4 activates the TIR-NB-LRR signal transducer for defense across cellular compartments^34–36^. Moreover, *EDS1* is an upstream regulator of the SA signaling pathway, and the *NPR4* gene codes for a protein that acts as an SA receptor^37,38^. Our results showed that the EDS1-, ABA-, and SA-mediated disease resistance pathways in Chrysanthemum have crucial roles in the early response to *A. alternata* infection. In *Arabidopsis*, *WRKY4* acts as a positive regulator of plant resistance to necrotrophic pathogens^39^. Among the WRKY target genes, *EDS1* and *NPR4* have been identified^40^. *CmWRKY4* showed high expression during the early infection stage, which indicates that Chrysanthemum *CmWRKY4* may act as an upstream regulator of *CmEDS1*, collectively contributing to the early defense response of Chrysanthemum leaves to *A. alternata* infection.

From the display of no symptoms to the expansion of necrotic lesions on Chrysanthemum leaves after *A. alternata* inoculation, ET plays a central role in defense. The expression levels of all the ET signal transduction pathway genes (*CmETR1*, *CmCTR1*, *CmEIN2*, and *CmERF2*) were upregulated during this transition. WGCNA also demonstrated that another major transcription factor involved in Ca^2+^ signaling, *CmWRKY33*, has a strong regulatory relationship with *CmERF2*. *CmWRKY33* and *CmERF2* were highly expressed in the stage of necrotic lesion formation at inoculation sites and may act as important regulators in response to *A. alternata* infection. Ca^2+^ participates in different types of cellular processes as a general secondary messenger in plants, with important roles of sensing a series of extracellular stimuli and responding quickly^28^. We also identified the gene encoding the calcium-binding EF-hand protein *CmCML45*, which is involved in the Ca^2+^ signaling pathway. In *Arabidopsis*, WRKY33 is a significant regulator involved in the transcriptional regulation of metabolic and hormone responses to *Botrytis cinerea* infection^41^. Compared with that which occurred in the resistant wild type, SA levels were elevated and JA-associated responses were downregulated in susceptible *WRKY33* mutants after infection with *B. cinerea*^42^. As reported, ERF2 appears to be positively controlled by WRKY33^43^. Both *ChiB* and *PDF1.2* can be activated by *ERF2*, which has been shown to lead to resistance to a number of pathogens^44,45^. In addition, *ACS2*, together with *ACS6*, is strongly correlated with the induction of ET, and WRKY33 can bind to the W-box region in the promoters of *ACS2* and *ACS6*, the process of which is directly associated with MPK-induced expression of *ACS2* and *ACS6*^46^. However, whether *CmWRKY33* is involved in ET signal transduction is still unknown. Our WGCNA showed that *CmCML45* and *CmWRKY33* have regulatory relationships with *CmERF2*, which was upregulated at 6 or 12 h after inoculation. *ERF2* is an important TF for the ET signal transduction pathway. ACC synthases (ACSs) convert S-adenosyl methionine to ACC, which is the direct precursor of ET^47^. The results of our study also showed that two ACS homologous genes (*DN61354c0_g1* and *DN93750c1_g1*) were upregulated at the early stage of *A. alternata* infection. Ca^2+^ influx depends on plasma membrane-localized CNGC family proteins, which are involved in the uptake of Ca^2+^ and initiate immune signaling in plant cells^31,48^. Two CNGC homologs (*DN96030c0_g1* and *DN93811c1_g2*) were also upregulated in the early stage. The above results show that Ca^2+^ signal transduction and the ET pathway play important roles in the early and middle stages of *A. alternata* infection in Chrysanthemum leaves.

In the late stage of *A. alternata* infection, necrotic lesions of Chrysanthemum leaves expanded, and a brown module was identified. However, no defense genes were identified among the hub genes. Interestingly, *CmDTX30* and *CmDTX32*, belonging to the MATE family, were identified hub genes in the brown module. Members of the MATE gene family play predominant roles in exporting toxins and other substrates^49,50^, and MATE proteins involved in the response to abiotic stress have been reported^51,52^. For example, *AtDTX50*, belonging to the MATE family, enhances drought tolerance in *Arabidopsis thaliana*^53^. However, the roles that members of the MATE gene family play in response to biotic stress remain unknown. In the brown module, *CmDTX30* and *CmDTX32* were highly upregulated at 24 or 36 h after inoculation, which indicated that some MATE family genes respond to *A. alternata* infection in the late stage. *Alternaria alternata* is a filamentous necrotrophic fungus that extracts nutrients from dead cells and produces different host-selective toxins that damage plant tissues^54,55^; seven pathogenic variants of *A. alternata* have been identified, which can cause disease in a variety of plants by producing host-specific toxins^2^. Based on the above studies, we speculate that Chrysanthemum leaves may transport toxins out of cells through multidrug resistance proteins, thereby reducing damage. Based on our results, we speculate that Chrysanthemum defends itself against *A. alternata* infection by reducing host-selective toxins via MATE family proteins. Additionally, ET signal transduction pathway genes exhibited peak upregulation at 24 hpi or 36 hpi, which indicated the important roles of ET in the late stage of the Chrysanthemum response to *A. alternata* infection.

Furthermore, 10 differentially expressed TFs were identified from the turquoise module, indicating that these proteins may play significant roles in the resistant cultivar ‘Jinba’. Among the TFs expressed in the ‘Jinba’ cultivar, a PTI6 homolog (*CmPTI6*; *DN80856c2_g1*) was highly expressed in the resistant cultivar ‘Jinba’ and differentially expressed after inoculation. PTI6, an important ERF transcription factor, specifically interacts with Pto kinase and binds to the GCC-box cis element, which is an important regulator in mediating the expression of pathogenesis-related (PR) genes^56^. We investigated the expression of PR homologous genes in Chrysanthemum, and the results showed that *PR3*-like genes (*DN75226c1_g1*, *DN87821c0_g1*, and *DN88995c0_g2*) were upregulated in ‘Jinba’ after *A. alternata* inoculation, with higher expression in ‘Jinba’ than in ‘Zaoyihong’ at most of the times assessed (Table S3). The results indicated that the PTI6 homolog and *PR3*-like genes play important roles in the response to *A. alternata* infection in ‘Jinba’.

**Fig. 7:**
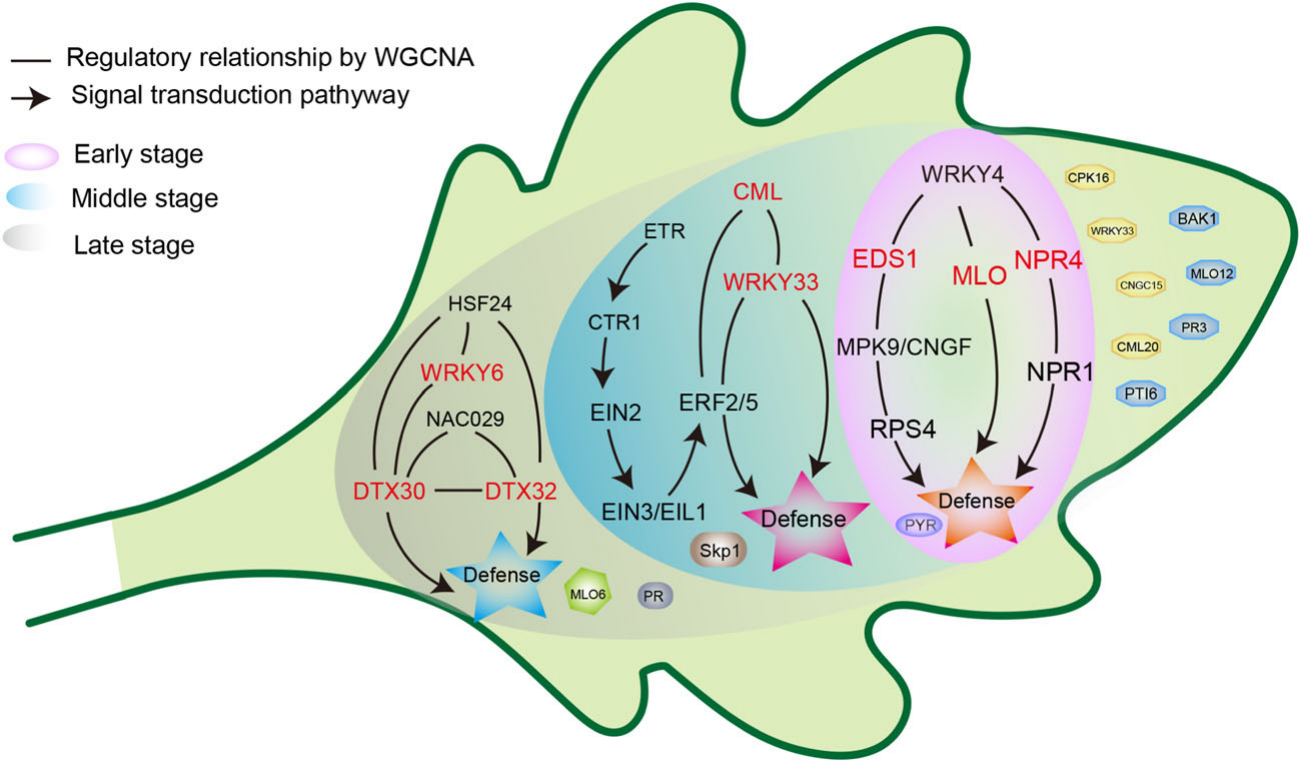
Gene expression model of the response of Chrysanthemum leaves to *A. alternata* infection. Different background colors represent the response of Chrysanthemum leaves to *A. alternata* infection at different stages, and the gene regulatory networks at different stages are shown in different color backgrounds. The correspondence between the background color and the infection stage is shown in the figure legend. Genes responding specifically to *A. alternata* infection in the resistant cultivar ‘Jinba’ are displayed in the green background of the leaf

Collectively, we mapped a model to elucidate the response of Chrysanthemum leaves to *A. alternata* infection at different stages and compared the model of the resistant cultivar ‘Jinba’ to that of the susceptible cultivar ‘Zaoyihong’ (Fig. 7). In the early stage, the calcium signal transduction pathway was activated in response to infection. With the transmission of calcium signals, more plant immune responses were activated, especially those involving the ET signaling pathway. Because *A. alternata* can produce a large number of toxins, we speculate that Chrysanthemum leaves may resist damage through MDR protein detoxification. In addition, we identified a series of hub genes including *PTI6,* and *PR3*-like genes, that were highly expressed specifically in response to *A. alternata* infection in the resistant cultivar ‘Jinba’. Our study systematically identified the immunity map of Chrysanthemum in response to *A. alternata* infection, revealed the defense strategies of Chrysanthemum in different infection periods, and added to our understanding of the plant immune response. The identification of the hub genes of Chrysanthemum lays a foundation for elucidating the mechanism of plant responses to pathogen infection at different periods. The hub genes identified from the resistant cultivar ‘Jinba’ provide a theoretical basis for breeding cultivars resistant to CBS.

## Methods

### Plant materials and *A. alternata* inoculation

Two Chrysanthemum cultivars (‘Jinba’ and ‘Zaoyihong’) and *A. alternata* were obtained from the Chrysanthemum Germplasm Resource Preserving Centre (Nanjing Agricultural University, China). Rooted seedlings were cultured in a 2:1 mixture of vermiculite and limestone. The seedlings were grown under a 16-h photoperiod with a day/night temperature of 25 °C and 18 °C, respectively, and the relative humidity was maintained at ~70%^57^. Mycelia of *A. alternata* were cultured in liquid media. Mycelia collected from 1 mL of liquid media were placed on a Chrysanthemum leaf. After inoculation, the Chrysanthemum seedlings were grown in the dark for 36 h. Chrysanthemum leaves were subsequently sampled at 1, 6, 12, 24, and 36 h post inoculation. Uninoculated leaves of Chrysanthemum were used as the control (0 h), which were collected at the beginning of the experiment before inoculation. Leaves infected by *A. alternata* collected at defined time points were used for RNA sequencing, with three replicates for each treatment.

### RNA extraction and sequencing

The total RNA from 36 samples was extracted according to previously reported methods^57^. All 36 libraries were constructed and sequenced using the Illumina platform at Beijing Novogene (Tianjin, China) to generate 150-nucleotide-long paired-end sequence reads. Clean reads were obtained from the raw reads by removing adapter sequences and low-quality reads. Chrysanthemum unigenes were acquired using Trinity (v2.4.0) for de novo assembly of the transcriptome^58^. The clean reads were then matched to the Chrysanthemum unigenes using RSEM software^59^. The frequency of individual reads was normalized to FPKM^60^.

### DEG identification and GO enrichment analysis

DESeq was used for DEG analysis^61^. The padj (adjusted *P*-value) provides a criterion to determine the *P*-value threshold in multiple tests and analyses by manipulating the adjusted *P*-value. In our study, differential expression was considered when the absolute value of the log2 induction ratios of the treated samples compared with the mock treatment or control samples (0 h) was ≥1.0 and when padj <0.05. To obtain comprehensive information on the function of the unigenes, seven major databases, including the nonredundant, nucleotide, Pfam, COG, Swiss-Prot, GO, and KEGG databases, were used for gene annotations. GO enrichment analysis of the DEG sets was performed using BINGO^62^. The *P*-value providing a criterion to determine enrichment for each represented GO term was calculated and corrected by the Benjamini and Hochberg false discovery rate (FDR). GO terms that showed corrected *P* values <0.05 were considered significantly enriched. Pathway enrichment analysis was then performed to identify the series of genes involved in the response of ‘Jinba’ and ‘Zaoyihong’ to *A. alternata* infection using MapMan (version 3.5.1)^63^.

### Coexpression network analysis for the construction of modules

The R package for WGCNA was used for coexpression network analysis to evaluate modules in which the genes showed high correlations^64^. An adjacency matrix was constructed based on normalized FPKM values. To identify modules related to phenotypic traits in the reconstruction network, the adjacency matrix was further converted to a topological overlap matrix (TOM) using the WGCNA package. After building the network, transcripts with similar expression patterns were classified into one module, and eigengenes (also called hub/key genes) for these modules were calculated. The TOM and degree were subsequently calculated; the top 20 genes according to degree were considered hub genes. Additionally, TOM >0.3 was considered highly correlated and strongly regulatory. Coexpression networks were visualized using Cytoscape software^65^.

### qPCR validation

Ten genes from different modules were selected for quantitative real-time polymerase chain reaction (qPCR) analysis. Gene-specific primer pairs of the selected genes were designed using the primer design website (https://www.yeastgenome.org/primer3) (Table S2), and qPCR assays were performed as previously described^66^. The Chrysanthemum *EF1α* gene was used as a reference. Transcript abundances are given as the means ± SEs of three replicates. Relative transcription levels were calculated by using the 2^−ΔΔCT^ method^67^. Three biological replicates were performed for each sample.

### Chrysanthemum *CmWRKY33.1* genetic transformation

Chrysanthemum plants were transformed with *Agrobacterium tumefaciens* strain EHA105 according to previously reported methods^68^. The coding region of *CmWRKY33.1* was cloned by polymerase chain reaction (PCR). According to the multiple cloning sites within the Chrysanthemum genetic transformation expression vector pENTR^TM^ 1A, two restriction endonuclease sites (*Sal*I and *Not*I) were selected for vector construction. *CmWRKY33.1* was inserted into pMDC43 by homologous recombination. The resultant pMDC43-*CmWRKY33.1* vector was then used for genetic transformation, and hygromycin was used to select stable transformants. DNA and RNA were isolated from the wild-type and transformant lines, which were used in PCR and qPCR experiments with constructed primers (Table S2) to identify the successfully transformed lines containing *CmWRKY33.1*.

## Supplementary information

Supplementary information
