# Supplementary material for: A temporal gene expression map of Chrysanthemum leaves infected with Alternaria alternata reveals different stages of defense mechanisms
Source: Hortic Res. 2020 Mar 1;7:23. doi: 10.1038/s41438-020-0245-0 (PMC7049303; doi:10.1038/s41438-020-0245-0)
Supplement: Supplementary file 1 — Supplementary information [file 41438_2020_245_MOESM1_ESM.docx]

**SUPPLEMENTARY INFORMATION FOR**

**A temporal gene expression map of Chrysanthemum leaves infected with *Alternaria alternata* reveals different stages of defense mechanisms**

Ye Liu^1,2^, Jingjing Xin^1,2^, Lina Liu^1^, Aiping Song^1^, Zhiyong Guan^1^, Weimin Fang^1^, Fadi Chen^1,*^

**Figure S1 Pearson correlation analyses between the 36 samples.**

**Figure S2 Heatmap analysis of DEGs related to plant-pathogen interaction and hormone signal transduction pathways in the leaves of Chrysanthemum ‘Jinba’ and ‘Zaoyihong’.**

**Figure S3 Heatmap of upregulated genes involved in hormone signal transduction.**

**Figure S4 MapMan-based visualization of an overview of the cellular response at 24 hpi in ‘Jinba’.**

**Figure S5 MapMan-based visualization of the biotic stress at 24 hpi in ‘Jinba’.**

**Figure S6 Verification of the expression of 10 select DEGs by quantitative real-time PCR (qPCR).**

**(a)** RNA sequencing data of 10 selected DEGs in ‘Jinba’ and ‘Zaoyihong’. The y-axis shows the log2-transformed fold change of each DEG.

**(b)** qPCR validation of 10 select DEGs in ‘Jinba’ and ‘Zaoyihong’. The y-axis shows the relative expression level of the 10 selected DEGs.

**Figure S7 Correlation analysis of gene expression ratios obtained by qPCR and RNA sequencing.**

**Table S1 Summary of sequencing reads after filtering.**

| Sample | Raw Reads | Clean Reads | Clean Bases | Error (%) | Q20(%) | Q30(%) | GC Content (%) |
| --- | --- | --- | --- | --- | --- | --- | --- |
| SM_0_1_1 | 24810240 | 23994171 | 3.6G | 0.03 | 97.54 | 93.37 | 42.99 |
| SM_0_1_2 | 24810240 | 23994171 | 3.6G | 0.03 | 97.16 | 92.16 | 42.95 |
| SM_0_2_1 | 26452609 | 25620180 | 3.84G | 0.03 | 97.61 | 93.39 | 42.85 |
| SM_0_2_2 | 26452609 | 25620180 | 3.84G | 0.04 | 94.86 | 87.32 | 42.74 |
| SM_0_3_1 | 24687467 | 23846216 | 3.58G | 0.03 | 97.51 | 93.25 | 42.66 |
| SM_0_3_2 | 24687467 | 23846216 | 3.58G | 0.03 | 97.29 | 92.46 | 42.63 |
| SM_1_1_1 | 23112098 | 22278931 | 3.34G | 0.03 | 97.51 | 93.27 | 42.4 |
| SM_1_1_2 | 23112098 | 22278931 | 3.34G | 0.03 | 96.93 | 91.68 | 42.35 |
| SM_1_2_1 | 26384738 | 25617157 | 3.84G | 0.03 | 97.55 | 93.36 | 42.29 |
| SM_1_2_2 | 26384738 | 25617157 | 3.84G | 0.03 | 96.64 | 91.03 | 42.22 |
| SM_1_3_1 | 22547670 | 21552150 | 3.23G | 0.03 | 97.35 | 93.02 | 43.93 |
| SM_1_3_2 | 22547670 | 21552150 | 3.23G | 0.03 | 96.41 | 90.65 | 43.87 |
| SM_6_1_1 | 25433194 | 24631920 | 3.69G | 0.03 | 97.56 | 93.35 | 43.17 |
| SM_6_1_2 | 25433194 | 24631920 | 3.69G | 0.03 | 97.07 | 91.95 | 43.12 |
| SM_6_2_1 | 22284493 | 21606977 | 3.24G | 0.03 | 97.4 | 92.97 | 43.01 |
| SM_6_2_2 | 22284493 | 21606977 | 3.24G | 0.03 | 97.12 | 92.04 | 42.96 |
| SM_6_3_1 | 23607821 | 23019931 | 3.45G | 0.03 | 97.53 | 93.23 | 43.25 |
| SM_6_3_2 | 23607821 | 23019931 | 3.45G | 0.03 | 96.92 | 91.63 | 43.21 |
| SM_12_1_1 | 23486558 | 22663306 | 3.4G | 0.03 | 97.58 | 93.4 | 43.45 |
| SM_12_1_2 | 23486558 | 22663306 | 3.4G | 0.03 | 97.15 | 92.13 | 43.41 |
| SM_12_2_1 | 24758937 | 24130102 | 3.62G | 0.03 | 97.57 | 93.39 | 44.11 |
| SM_12_2_2 | 24758937 | 24130102 | 3.62G | 0.03 | 97.04 | 91.93 | 44.05 |
| SM_12_3_1 | 26507444 | 25672779 | 3.85G | 0.03 | 97.51 | 93.32 | 42.81 |
| SM_12_3_2 | 26507444 | 25672779 | 3.85G | 0.03 | 96.83 | 91.49 | 42.73 |
| SM_24_1_1 | 24327809 | 23616144 | 3.54G | 0.03 | 97.57 | 93.5 | 47.02 |
| SM_24_1_2 | 24327809 | 23616144 | 3.54G | 0.03 | 96.66 | 91.22 | 46.94 |
| SM_24_2_1 | 21691448 | 21126987 | 3.17G | 0.03 | 97.66 | 93.56 | 44.96 |
| SM_24_2_2 | 21691448 | 21126987 | 3.17G | 0.03 | 96.64 | 91.03 | 44.87 |
| SM_24_3_1 | 23452985 | 22801822 | 3.42G | 0.03 | 97.63 | 93.53 | 44.4 |
| SM_24_3_2 | 23452985 | 22801822 | 3.42G | 0.03 | 96.5 | 90.8 | 44.32 |
| SM_36_1_1 | 24328798 | 23602327 | 3.54G | 0.03 | 97.52 | 93.34 | 45.82 |
| SM_36_1_2 | 24328798 | 23602327 | 3.54G | 0.03 | 96.71 | 91.29 | 45.73 |
| SM_36_2_1 | 21168142 | 20599621 | 3.09G | 0.03 | 97.62 | 93.53 | 45.94 |
| SM_36_2_2 | 21168142 | 20599621 | 3.09G | 0.03 | 97.37 | 92.7 | 45.91 |
| SM_36_3_1 | 21172204 | 20601703 | 3.09G | 0.03 | 97.57 | 93.42 | 44.4 |
| SM_36_3_2 | 21172204 | 20601703 | 3.09G | 0.03 | 96.64 | 91.1 | 44.33 |
| ZYH_0_1_1 | 22670257 | 21982786 | 3.3G | 0.03 | 97.52 | 93.37 | 44.42 |
| ZYH_0_1_2 | 22670257 | 21982786 | 3.3G | 0.03 | 97.16 | 92.29 | 44.35 |
| ZYH_0_2_1 | 24429846 | 23885187 | 3.58G | 0.03 | 97.69 | 93.6 | 44.27 |
| ZYH_0_2_2 | 24429846 | 23885187 | 3.58G | 0.03 | 97.26 | 92.36 | 44.21 |
| ZYH_0_3_1 | 24831187 | 24198064 | 3.63G | 0.03 | 97.71 | 93.63 | 44.41 |
| ZYH_0_3_2 | 24831187 | 24198064 | 3.63G | 0.03 | 96.77 | 91.31 | 44.33 |
| ZYH_1_1_1 | 22253611 | 21616935 | 3.24G | 0.03 | 97.61 | 93.46 | 44.2 |
| ZYH_1_1_2 | 22253611 | 21616935 | 3.24G | 0.03 | 97.06 | 91.96 | 44.14 |
| ZYH_1_2_1 | 24486301 | 23851712 | 3.58G | 0.03 | 97.57 | 93.35 | 42.91 |
| ZYH_1_2_2 | 24486301 | 23851712 | 3.58G | 0.03 | 96.78 | 91.37 | 42.85 |
| ZYH_1_3_1 | 24323036 | 23676336 | 3.55G | 0.03 | 97.55 | 93.32 | 43.43 |
| ZYH_1_3_2 | 24323036 | 23676336 | 3.55G | 0.03 | 96.89 | 91.59 | 43.39 |
| ZYH_6_1_1 | 25192668 | 24577488 | 3.69G | 0.03 | 97.65 | 93.58 | 44.45 |
| ZYH_6_1_2 | 25192668 | 24577488 | 3.69G | 0.03 | 96.54 | 90.85 | 44.36 |
| ZYH_6_2_1 | 20736797 | 20123952 | 3.02G | 0.03 | 97.65 | 93.5 | 44.18 |
| ZYH_6_2_2 | 20736797 | 20123952 | 3.02G | 0.03 | 97.06 | 91.89 | 44.12 |
| ZYH_6_3_1 | 24423734 | 23787661 | 3.57G | 0.03 | 97.54 | 93.2 | 43.2 |
| ZYH_6_3_2 | 24423734 | 23787661 | 3.57G | 0.03 | 97.18 | 92.15 | 43.16 |
| ZYH_12_1_1 | 26898544 | 26206591 | 3.93G | 0.03 | 97.66 | 93.56 | 44.51 |
| ZYH_12_1_2 | 26898544 | 26206591 | 3.93G | 0.03 | 97.18 | 92.24 | 44.46 |
| ZYH_12_2_1 | 24489757 | 23661659 | 3.55G | 0.03 | 97.63 | 93.57 | 46.69 |
| ZYH_12_2_2 | 24489757 | 23661659 | 3.55G | 0.03 | 96.7 | 91.25 | 46.6 |
| ZYH_12_3_1 | 25073134 | 24191010 | 3.63G | 0.03 | 97.59 | 93.47 | 44.09 |
| ZYH_12_3_2 | 25073134 | 24191010 | 3.63G | 0.03 | 97.03 | 91.87 | 44.04 |
| ZYH_24_1_1 | 24301244 | 23598119 | 3.54G | 0.03 | 97.6 | 93.45 | 44.24 |
| ZYH_24_1_2 | 24301244 | 23598119 | 3.54G | 0.03 | 96.79 | 91.37 | 44.17 |
| ZYH_24_2_1 | 24097762 | 23396107 | 3.51G | 0.03 | 97.68 | 93.62 | 44.24 |
| ZYH_24_2_2 | 24097762 | 23396107 | 3.51G | 0.03 | 96.93 | 91.68 | 44.18 |
| ZYH_24_3_1 | 25910260 | 25021539 | 3.75G | 0.03 | 97.54 | 93.29 | 47.29 |
| ZYH_24_3_2 | 25910260 | 25021539 | 3.75G | 0.03 | 97.04 | 91.98 | 47.24 |
| ZYH_36_1_1 | 24375450 | 23579474 | 3.54G | 0.03 | 97.6 | 93.44 | 44.35 |
| ZYH_36_1_2 | 24375450 | 23579474 | 3.54G | 0.03 | 97.28 | 92.43 | 44.31 |
| ZYH_36_2_1 | 25435265 | 24711667 | 3.71G | 0.03 | 97.7 | 93.63 | 44.32 |
| ZYH_36_2_2 | 25435265 | 24711667 | 3.71G | 0.03 | 96.67 | 91.11 | 44.25 |
| ZYH_36_3_1 | 20539470 | 19914813 | 2.99G | 0.03 | 97.61 | 93.44 | 44.87 |
| ZYH_36_3_2 | 20539470 | 19914813 | 2.99G | 0.03 | 97.08 | 91.99 | 44.82 |

**Table S2 Primer sequences used in this study.**

| Primer | Sequence (5’-3’) | Relevant characteristics |
| --- | --- | --- |
| CmEF1α-F  CmEF1α-R | TCAGGTCATCATCATGAACCA  AAGAGGTGGGTACTCAGCAAA | PCR primers for amplification of the reference gene Cm*EF1α* in quantitative real-time PCR assays |
| DN69937c2_g1-F  DN69937c2_g1-R | AATGTTTCCATCCCGTTTTCG  ACGTGTGTTTTGGACAATTTGG | PCR primers for amplification of the *CmCML45* in quantitative real-time PCR assays |
| DN63096c1_g1-F  DN63096c1_g1-R | CCAAGCCTCAAAATGCCAAA  CGAATGATGCCGAAGAATCC | PCR primers for amplification of the *CmWRKY33* in quantitative real-time PCR assays |
| DN90778c3_g1-F  DN90778c3_g1-R | TGCCAATAAACCCTCCGATG  AGTTGCTTATGATATGGCGGC | PCR primers for amplification of the *CmERF2* in quantitative real-time PCR assays |
| DN82662c0_g1-F  DN82662c0_g1-R | TGAGATCAATCGCCGAAAAAC  CATCCCCGGAACTAGAACCA | PCR primers for amplification of the *CmHSF24* in quantitative real-time PCR assays |
| DN73063c1_g1-F  DN73063c1_g1-R | CCTGAGAAAGCGGAATTTGG  CCGAGACAGCTGCCCTATTG | PCR primers for amplification of the *CmNAC029* in quantitative real-time PCR assays |
| DN77454c0_g1-F  DN77454c0_g1-R | GATCGACGATGAAGCCAACA  CACTTGGCCCGACTTTTTGT | PCR primers for amplification of the *CmMLO6* in quantitative real-time PCR assays |
| DN77779c0_g1-F  DN77779c0_g1-R | TCTGGCGAGGTAGATGACA  GTACCATAGATCCATGTCGTTCA | PCR primers for amplification of the *CmMYB15* in quantitative real-time PCR assays |
| DN81968c0_g1-F  DN81968c0_g1-R | TGACAGGGGATATGGATAGGAA  CTCATTTTCATCCGACTTCAAGT | PCR primers for amplification of the *CmDTX24* in quantitative real-time PCR assays |
| DN87341c1_g3-F  DN87341c1_g3-R | GTTTGCTGATGTGGCTGGAG  AGCGTCTTCCCAGTCCCG | PCR primers for amplification of the *CmFTSH* in quantitative real-time PCR assays |
| DN93367c1_g4-F  DN93367c1_g4-R | GGTTGTTCTGTTACCGGTGT  AGCGACCTAACCATCTCTCA | PCR primers for amplification of the *CmVTE3* in quantitative real-time PCR assays |
| CmWRKY33.1-ID-F1  CmWRKY33.1-ID-R1 | CTGTCCACACAATCTGCC  GAGCGAACCTAGATCCACGAA | PCR primers for amplification of pMDC43-*CmWRKY33.1* vector in transformation lines |
| CmWRKY33.1-ID-F2  CmWRKY33.1-ID-R2 | GGCGATTAAGCCTATGGCAAT  ACCCATCTCATAAATAAC | PCR primers for amplification of pMDC43-*CmWRKY33.1* vector in transformation lines |
| CmWRKY33.1-F  CmWRKY33.1- R | TTCAGTCGACATGAGTTCCACAGCCATACA  GAGTGCGGCCGCGAAAATAGCAACGACTCCAAGA | PCR primers for amplification of *CmWRKY33.1* sense fragment for vector construction |
| CmWRKY33.1-QRT-F  CmWRKY33.1-QRT-R | GGCGATTAAGCCTATGGCAAT  GAGCGAACCTAGATCCACGAA | PCR primers for amplification of *CmWRKY33.1* in quantitative real-time PCR assays |

**Table S3 FPKM of differentially expressed genes.**

| gene id | Description | SM_0 | SM_1 | SM_6 | SM_12 | SM_24 | SM_36 | ZYH_0 | ZYH_1 | ZYH_6 | ZYH_12 | ZYH_24 | ZYH_36 |
| --- | --- | --- | --- | --- | --- | --- | --- | --- | --- | --- | --- | --- | --- |
| DN75226c1_g1 | PR3-like | 2.55 | 21.9933333 | 501.693333 | 712.683333 | 6804.20333 | 3713.78667 | 1.45666667 | 32.8166667 | 106.143333 | 1346.17333 | 5698.89 | 1618.63333 |
| DN87821c0_g1 | PR3-like | 125.856667 | 1171.17333 | 1085.74667 | 1104.29333 | 2405.59333 | 854.183333 | 63.6833333 | 1700.76 | 998.95 | 1670.23667 | 1633.09 | 385.783333 |
| DN88995c0_g2 | PR3-like | 15.0533333 | 46.0466667 | 52.1366667 | 46.9966667 | 52.3366667 | 22.8366667 | 10.96 | 35.9233333 | 29.02 | 67.5066667 | 67.5633333 | 33.9866667 |
| DN82887c0_g1 | MATE family | 105.003333 | 109.496667 | 169.07 | 273.86 | 85.7566667 | 139.67 | 11.6933333 | 15.3866667 | 23.7633333 | 16.1933333 | 19.0533333 | 17.0466667 |
| DN90910c1_g1 | MATE family | 32.4533333 | 29.27 | 49.22 | 50.38 | 25.6966667 | 31.8266667 | 26.84 | 20.2 | 26.94 | 18.1966667 | 15.43 | 24.8566667 |
| DN94014c1_g1 | MATE family | 51.7066667 | 96.4666667 | 54.12 | 50.3433333 | 62.68 | 48.2833333 | 17.86 | 47.15 | 18.49 | 30.0533333 | 34.5466667 | 29.2566667 |
| DN95310c2_g2 | MATE family | 42.78 | 43.6 | 45.9266667 | 43.7333333 | 24.5766667 | 37.9 | 25.41 | 30.75 | 31.8766667 | 21.7133333 | 26.2166667 | 32.9866667 |
| DN91413c1_g3 | ABCA3 family | 21.5833333 | 48.1566667 | 145.836667 | 134.816667 | 101.046667 | 69.3166667 | 8.66666667 | 22.3433333 | 24.1433333 | 37.8233333 | 45.63 | 31.68 |
